# COVID-19 in otolaryngologists: a cross-sectional multicenter study

**DOI:** 10.1016/j.bjorl.2021.06.007

**Published:** 2021-07-19

**Authors:** Fabrício Scapini, José Faibes Lubianca Neto, Roberto Dihl Angeli, Rita Carolina Pozzer Krumenauer, Ingrid Wendland Santanna, Luciana Pimentel Oppermann, Nedio Atolini Junior, Camila Degen Meotti, Caroline Catherine Lacerda Elias, Lilcia Helena de Britto Medeiros, Renato Roithmann, Clarissa Delpizzo Castagno, Adriana de Carli, Eduardo Homrich Granzotto, Nedio Steffen, Gerson Schulz Maahs

**Affiliations:** a Universidade Federal de Santa Maria (UFSM), Santa Maria, RS, Brazil; b Universidade Federal de Ciências da Saúde de Porto Alegre (UFCSPA), Porto Alegre, RS, Brazil; c Universidade Luterana do Brasil (ULBRA), Canoas, RS, Brazil; d Associação Gaúcha de Otorrinolaringologia e Cirurgia Cérvico-facial (ASSOGOT-CCF), Porto Alegre, RS, Brazil; e Hospital Nossa Senhora da Conceição, de Porto Alegre (GHC), Porto Alegre, RS, Brazil; f Universidade Federal da Fronteira Sul (UFFS), Passo Fundo, RS, Brazil; g Universidade Federal do Rio Grande do Sul (UFRGS), Porto Alegre, RS, Brazil; h Universidade de Caxias do Sul (UCS), Caxias do Sul, RS, Brazil; i Pontifícia Universidade Católica do Rio Grande do Sul (PUCRS), Porto Alegre, RS, Brazil

**Keywords:** COVID-19, SARS-CoV-2, Otolaryngologists, Seroprevalence, Health-care workers

## Abstract

**Introduction:**

The nose and throat are areas of high viral load, which could place otolaryngologists at an even higher risk for COVID-19 than other health-care workers.

**Objective:**

To investigate the prevalence of antibodies against SARS-CoV-2 in otorhinolaryngologists in southern Brazil, its relationship to demographic data, professional practice and reported symptoms of COVID-19, and compare it with official data on other health-care workers of the state and the general population in the same period.

**Methods:**

In this cross-sectional multicenter study, otolaryngologists actively practicing officially registered in Rio Grande do Sul were screened for IgM and IgG antibodies against SARS-CoV-2 from August 1 to September 15, 2020. A questionnaire was also applied.

**Results:**

We screened 358 (80.1%) of 447 actively practicing otolaryngologists (195 [54.5%] male; mean [SD] age, 47.77 [13.57] years; range, 26–84 years). Twenty-three were positive for IgM and/or IgG (6.4%). This result was significantly associated with reports of infected household contacts (19/315 negatives and 8/23 positives; *p* < 0.001). From 23 seropositive participants, 14 were asymptomatic (60.9%; *p* < 0.001). There were no significant associations between seroconversion and age, sex, number of patient appointments and surgical procedures, workplace (hospital or private practice), patients with or without respiratory symptoms, or level of personal protective equipment used. The rate of COVID-19 in all health-care workers in the state was 7.69% at the end of the same period. Data from state government seroprevalence was 5.26 (risk ratio [RR]; 95% CI 3.27–8.45) and 4.66 (RR; 95% CI 2.93–7.43) times higher in otolaryngologists than in the general population in August and September, respectively.

**Conclusion:**

Otolaryngologists had a higher seroconversion rate than the general population. Using personal protective equipment, the level of occupational exposure did not result in higher rates of infection than other health-care workers, but the presence of infected household contacts was associated with higher rates of seroconversion.

## Highlights

• Otolaryngologists have a high risk of contamination by SARS-CoV-2.
• The pandemic had a negative impact on ENT care.
• Infections by SARS-CoV-2 were similar between ENTs and other health-care workers.
• Personal protective equipment is essential for healthcare professionals.

## Introduction

Soon after emergence of the COVID-19 outbreak, caused by severe acute respiratory syndrome coronavirus 2 (SARS-CoV-2), it became evident that health-care workers were particularly vulnerable to SARS-CoV-2 infection,^1^, ^2^ including not only frontline personnel, such as nurses and other critical-care staff, but also general practitioners. Preliminary data from Italy and the United States indicated that 10% and 18% of all individuals who tested positive for SARS-CoV-2, respectively, were health-care workers.^3^, ^4^

As the nose and nasopharynx serve as a reservoir for high concentrations of SARS-CoV-2,^5^ the first reports early on in the pandemic noted that otolaryngologists were likely to be highly predisposed to SARS-CoV-2 infection, since they have greater contact with upper airway mucosa during endoscopic and invasive procedures.^6^ An international survey, including the 30 countries with the highest incidence of COVID-19 on April 20, 2020, reported 361 infected otolaryngologists. However, the study design limits the interpretation of these results.^7^

We aimed to investigate the prevalence of IgM and IgG antibodies against SARS-CoV-2 in otolaryngologists actively practicing in Rio Grande do Sul, the southernmost state of Brazil. Additionally, we examined associations between positive test results and variables related to professional practice, symptoms, and infection in household contacts, which are believed to be important factors in SARS-CoV-2 transmission. To our knowledge, there are no studies that have objectively tested a representative sample of a medical specialty at increased risk of contracting COVID-19, which is the case of otolaryngology.

## Methods

This cross-sectional multicenter study was led by Universidade Federal de Santa Maria (UFSM) and conducted in partnership with the following institutions: Universidade Federal de Ciências da Saúde de Porto Alegre (UFCSPA), Universidade Federal do Rio Grande do Sul (UFRGS), Pontifícia Universidade Católica do Rio Grande do Sul (PUC-RS), Universidade Luterana do Brasil (ULBRA), Universidade Federal da Fronteira Sul (UFFS), Grupo Hospitalar Conceição (GHC), Universidade de Caxias do Sul (UCS), and the Rio Grande do Sul Association for Otolaryngology (ASSOGOT). All institutions are located in Rio Grande do Sul. The study sample consisted of otolaryngologists actively practicing in the state who were identified through a public-domain list of practicing otolaryngologists registered with the Brazilian Federal Medical Council (available at http://portal.cfm.org.br/index.php?option=com_medicos&Itemid=59). The study was approved by UFSM Research Ethics Committee and the local ethics committees at all participating institutions (CAEE 35517220.5.1001.5346). Written informed consent was obtained from each study participant.

All active/registered otolaryngologists were contacted by telephone and/or email. Those who agreed to participate were recorded as “agreed” and received an envelope containing the consent form (with a detailed explanation of the objectives, methodology, and expected results of the research), the demographic, professional practice, and symptoms questionnaire (DPPSQ), which covered COVID-19-related symptoms that may have been experienced in the previous 30 days, a confidentiality agreement, a results slip, and a chromatographic immunoassay kit. The envelopes were preferably delivered in person by an author who lived in the same region as the participant and who also administered the tests. If the participant lived in a town far from any of the participating centers, the envelope was delivered by an ASSOGOT staff member or mailed to the participant. In these cases, the tests were self-administered by the participants and returned in a prepaid self-addressed envelope. The authors produced a video tutorial on the testing procedures, which was emailed to all participants. Sealed envelopes were returned to the researchers in charge of data collection, thus ensuring data confidentiality. Data were collected from August 1 to September 15.

### Eligibility criteria

Otolaryngologists were included if they (1) were duly registered with the Brazilian Federal Medical Council at the time of the survey, (2) were actively practicing in the state through the Brazilian Unified Health System or privately (including patient appointments and/or surgical procedures) prior to the pandemic declaration on March 11, 2020, (3) returned a signed form consenting to participate in the study and to individually provided a sample for a rapid IgM-IgG combined antibody test for SARS-CoV-2 infection diagnosis, and (4) returned a completed questionnaire. Otolaryngologists who (1) were inactive or practicing outside the state in the period immediately prior to the World Health Organization (WHO) pandemic declaration, (2) could not be contacted for invitation and/or explanations, (3) were no longer living in the state, (4) did not return the questionnaire or returned an incomplete questionnaire, or (5) failed to meet the deadline for serological testing for SARS-CoV-2 antibodies were excluded.

### Demographic, professional practice, and symptoms questionnaire (DPPSQ)

The DPPSQ is a 13-item questionnaire covering demographic information (age, sex, and town where participants practice), variables related to professional practice, and COVID-19-related symptoms. Three questions gathered data on the average number of patient appointments per week in the past 4 weeks, whether this number had reduced in relation to the period prior to the pandemic, and the average number of surgical procedures performed in the past 4 weeks. Four questions were related to the workplace (hospital or private practice), personal protective equipment (PPE), and number of patients seen with acute respiratory symptoms. The participants were also asked whether they had experienced respiratory symptoms in the past 4 weeks, whether any of their household contacts had been diagnosed with COVID-19, and whether they had undergone any SARS-CoV-2 testing prior to the study (if so, they were asked to inform us of the test results).

### Chromatographic immunoassay

The COVID-19 IgG/IgM ECO Test (ECO Diagnostica LTDA™, Brazil) was used in the study. Each kit contains one test device (cassette), a 20 µL disposable capillary pipette, a sterile disposable lancet, a flask containing buffer solution for dilution, a disposable wet wipe with 70% alcohol, and a post-puncture dressing. Capillary whole blood was collected aseptically from a fingertip. According to information provided by the manufacturer, the test has a sensitivity of 94.51% and a specificity of 95.74% in the detection of SARS-CoV-2 at least 7 days after the onset of symptoms.

### Statistical analysis

For descriptive analysis, quantitative variables were expressed as mean (SD) or median (minimum and maximum values) and qualitative variables were expressed as absolute and relative frequencies. Student’s *t* test was used to compare means between 2 groups. When the assumption of normality of distribution was violated, the Mann–Whitney nonparametric test was used. Homogeneity between proportions was tested using the Chi-Square test or Fisher’s exact test. Data were analyzed in SPSS for Windows, version 17.0 (Chicago, IL, United States), and the significance level was set at 5% for all analyses. We were unable to perform multivariate logistic regression analysis because there were many structural zeros. The sensitivity, specificity, and accuracy of the serological test was evaluated against the gold standard (self-reported RT-PCR-positive SARS-CoV-2 test). Results were reported following STROBE statement for observational studies.

## Results

**Figure 1:**
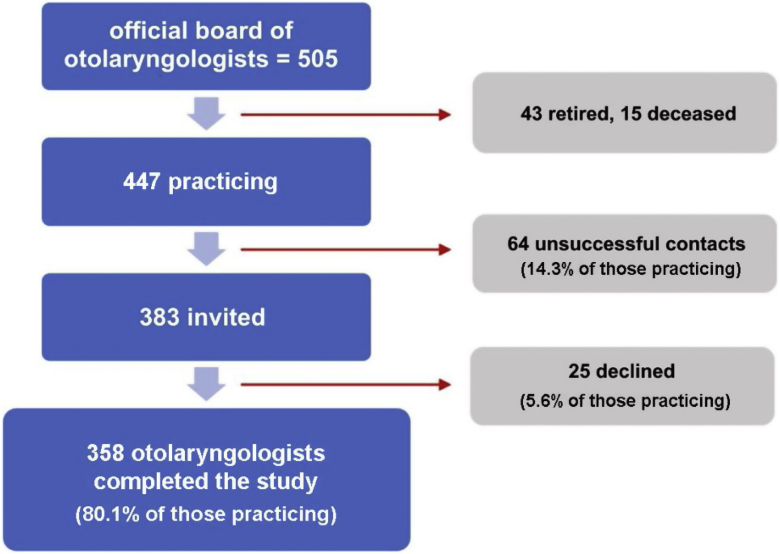
Flowchart illustrating participant recruitment.

Of 505 otolaryngologists registered with the Brazilian Federal Medical Council, 447 were eligible for inclusion (excluding those who had retired or died). Of these, 358 were included, accounting for 80.1% of all actively practicing in the state. In total, 306 were tested directly by the researchers and 52 received the tests by mail or by ASSOGOT staff for self-testing. The recruitment process is illustrated in Fig. 1.

Twenty-three (6.4%) otolaryngologists had positive IgM or IgG, or both, antibodies against SARS-CoV-2. Of these, six were positive for IgM and IgG, seven were positive for IgM only and 10 were positive for IgG only. The results are shown in Table 1.

**Table 1:** Associations between presence of antibodies against SARS-CoV-2 and variables related to demographics, professional practice, and symptoms in otolaryngologists from Rio Grande do Sul, Brazil.

Variable	IgM and/or IgG		*p*
	Negative (N = 335)	Positive (N = 23)	
**Age (years)**	47.90 ± 13.63	45.78 ± 12.82	0.470^a^
**Sex**			0.508^b^
**Male**	184 (54.9%)	11 (47.8%)	
**Female**	151 (45.1%)	12 (52.2%)	
**Number of patient appointments (per week)**			0.907^b^
<10	36 (10.7%)	2 (8.7%)	
10–40	153 (45.7%)	10 (43.5%)	
>40	146 (43.6%)	11 (47.8%)	
**Number of surgeries (in the last 4 weeks)**	3.06 ± 4.75	1.39 ± 1.50	0.243^c^
	**Median = 1.00**	**Median = 1.00**	
**Reduced workload during the pandemic**			0.498^d^
<30%	91 (27.2%)	3 (13.0%)	
30%–60%	201 (60.0%)	17 (73.9%)	
>60%	39 (11.6%)	3 (13.0%)	
**Temporarily inactive**	4 (1.2%)	0 (0.0%)	
**Workplace**			0.939^b^
Hospital	14 (4.2%)	1 (4.3%)	
Private practice	216 (64.5%)	14 (60.9%)	
Both	105 (31.3%)	8 (34.8%)	
**Type of hospital**			
Public, non-COVID-19	20 (16.8%)	0 (0.0%)	0.353^d^
Public, COVID-19	59 (49.6%)	5 (55.6%)	1.000^d^
Private, non-COVID-19	28 (23.5%)	2 (22.2%)	1.000^d^
Private, COVID-19	54 (45.4%)	3 (33.3%)	0.730^d^
**% of patients seen**			0.194^d^
0%–25%	221 (66.0%)	14 (60.9%)	
25%–50%	73 (21.8%)	5 (21.7%)	
50%–75%	17 (5.1%)	4 (17.4%)	
75%–100%	5 (1.5%)	0 (0.0%)	
Not practicing	19 (5.7%)	0 (0.0%)	
**Personal protective equipment**			0.775^b^
N95 mask + gloves	73 (22.1%)	3 (13.0%)	
N95 mask + gloves + goggles	81 (24.5%)	6 (26.1%)	
Mask + gloves + face shield	94 (28.4%)	7 (30.4%)	
Mask + gloves + face shield + apron	83 (25.1%)	7 (30.4%)	
**Reported RT-PCR**			<0.001^d^
Positive	0 (0.0%)	6 (26.1%)	
Negative	46 (13.8%)	1 (4.3%)	
Not taken	288 (86.2%)	16 (69.6%)	
**Reported IgM**			0.051^d^
Positive	0 (0.0%)	1 (4.3%)	
Negative	86 (25.7%)	7 (30.4%)	
Not taken	249 (74.3%)	15 (65.2%)	
**Reported IgG**			<0.001^d^
Positive	1 (0.3%)	4 (17.4%)	
Negative	87 (26.0%)	5 (21.7%)	
Not taken	247 (73.7%)	14 (60.9%)	
**Asymptomatic**			<0.001^d^
Yes	298 (89.0%)	14 (60.9%)	
No	37 (11.0%)	9 (39.1%)	
**Infected household contact**			<0.001^d^
None	315 (94.3%)	15 (65.2%)	
Spouse	10 (3.0%)	8 (34.8%)	
Child	4 (1.2%)	0 (0.0%)	
Sibling	1 (0.3%)	0 (0.0%)	
Other	4 (1.2%)	0 (0.0%)	

Values are presented as mean ± standard deviation or N (%).

a Descriptive probability level according to Student’s *t* test.

b Descriptive probability level according to Chi-Square test.

c Descriptive probability level according to Mann–Whitney nonparametric test.

d Descriptive probability level according to Fisher’s exact test.

Seropositive participants had a significantly higher rate of self-reported positive RT-PCR (6/6 respectively; *p* < 0.001) and IgG (4/5; *p* < 0.001) test results, with a trend toward significance for positive IgM test results (1/1; *p* = 0.051), and a higher rate of symptomatic cases and infected household contacts than seronegative participants. There were no statistically significant differences between the two groups in any of the other variables analyzed.

**Figure 2:**
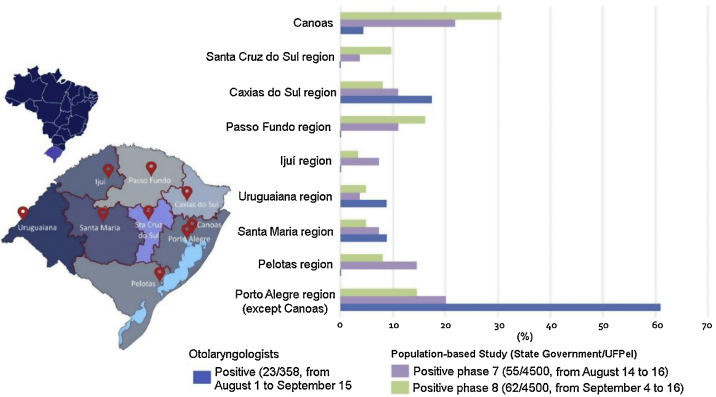
Distribution of cases reported in the present study and cases from a population-based study conducted by the State Government/UFPel per region of Rio Grande do Sul, Brazil.^19^, ^20^

Fig. 2 illustrates COVID-19 groups per region within the state. No significant differences (Fisher’s exact test *p* = 0.142) were found between participants who were IgM and/or IgG positive in relation to the region of the state where they practiced otolaryngology (Fig. 2).

Forty-six otolaryngologists reported COVID-19-related symptoms in the 4 weeks preceding testing. Table 2 shows the distribution of symptoms and their associations with positive antibody test results. Reports of headaches, fatigue, myalgia, and anosmia/ageusia were significantly more common in seropositive than in seronegative participants.

**Table 2:** Symptoms in the 4 weeks prior to the study and presence of antibodies against SARS-CoV-2 in 46 symptomatic otolaryngologists.

Symptom	IgM and/or IgG positive		*p*^a^
	No (N = 335)	Yes (N = 23)	
	n	n	
Headaches	16 (4.8%)	4 (17.4%)	0.032
Sore throat	17 (5.1%)	2 (8.7%)	0.349
Fatigue	12 (3.6%)	4 (17.4%)	0.014
Myalgia	9 (2.7%)	3 (13.0%)	0.035
Dry cough	10 (3.0%)	2 (8.7%)	0.176
Diarrhea	6 (1.8%)	1 (4.3%)	0.374
Anosmia/ageusia	0	6 (26.1%)	<0.001
Fever	3 (0.9%)	1 (4.3%)	0.234
Skin rash	3 (0.9%)	0	1
Shortness of breath	3 (0.9%)	0	1
Chest pain	2 (0.6%)	1 (4.3%)	0.181
Conjunctivitis	1 (0.3%)	0	1

a Descriptive probability level according to Fisher’s exact test.

The self-report of positive RT-PCR served as the gold standard in relation to the sensitivity and negative predictive value of the rapid qualitative antibody test used in our study (Table 2). The results showed that 100% of the participants with a positive test by RT-PCR also tested positive for IgG or IgM antibodies (100% sensitivity). The proportion of all correct test results, whether positive or negative, was 98.1% (98.1% accurate). Regarding specificity, 97.9% of participants with a negative test by RT-PCR did not present a positive test for IgG or IgM antibodies. These results are shown in Table 3.

**Table 3:** Performance of the IgM-IgG combined antibody test for SARS-CoV-2 used in the study.

		PCR		Total
		Positive	Negative	
IgG/IgM	Positive	6	1	7
	Negative	0	46	46
Total		6	47	53

Sensitivity = 6/6 × 100 = 100%.

Specificity = 46/47 × 100 = 97.9%.

Positive predictive value = 6/7 × 100 = 85.7%.

Negative predictive value = 46/46 × 100 = 100%.

Accuracy = 52/53 × 100 = 98.1%.

## Discussion

The first death of a physician associated with SARS-CoV-2 was of an otolaryngologist, on February 25, 2020, in Wuhan, just as during the SARS pandemic in 2003, when the first death was also of an otolaryngologist in Hong Kong.^13^ A report with the director of an intensive care unit in mid-March rapidly publicized his declaration that ophthalmologists and otolaryngologists were the medical specialists most affected by the novel coronavirus.^14^ A frequently-cited letter published on April 15 stated that, according to informal reports from colleagues in Iran, around 20 otolaryngologists had been hospitalized, a further 20 were symptomatic, and one young specialist had died. The same publication claimed that 14 health-care workers were infected during an endoscopic skull base surgical operation in Wuhan.^15^ However, it was later pointed out that none of the physicians who took part in the operation were infected and that none of the four physicians from the neurosurgery department who were infected had been in contact with the patient.^16^ After these reports, concerns became more focused on PPE precautions and the risk of infection among health-care workers, especially in otolaryngologists, who are very often involved in procedures that generate aerosols.

Rio Grande do Sul is Brazil’s southernmost state and has an estimated population of 11.4 million inhabitants.^8^ Data from the State Health Department show that, on August 1, 2020 (the first day of our data collection period), the state had 91,471 confirmed cases, with 2146 deaths, reaching 185,376 cases and 4370 deaths on September 15, 2020 (the last day of our data collection period). The apparent lethality was 2.36%. Health-care workers accounted for 7.69% of the confirmed cases (14,254 cases).^9^

The present study found a seroprevalence of IgM and/or IgG antibodies against SARS-CoV-2 of 6.4% in the 358 participating otolaryngologists. Nguyen et al., using a smartphone application to obtain prospective data from a cohort of just over 2 million participants, of whom 4.7% were health-care workers, found, after adjustments, a hazard ratio of 3.40 (95% CI 3.37–3.43) for risk of reporting a positive COVID-19 test.^10^

Data from a population-based study conducted in Rio Grande do Sul by the state government and Universidade Federal de Pelotas (UFPel), with similar methodology and data collection periods (from August 14 to 16 and from September 4 to 6), revealed an estimated prevalence of 1.22% and 1.38%, respectively, for antibodies against SARS-CoV-2 in the general population of the state.^11^, ^12^ Compared to the present findings, seroprevalence was 5.26 (RR; 95% CI 3.27–8.45) times higher in otolaryngologists than in the general population of the state in August and 4.66 (RR; 95% CI 2.93–7.43) times higher in September.

Most seropositive otolaryngologists were either asymptomatic or had mild symptoms, with no need for hospitalization. To date (January 10, 2021), at least 465 physicians (including 5 otolaryngologists) have died in Brazil and 4 have died in Rio Grande do Sul, none of whom were otolaryngologists.^17^

Forty-six participants had COVID-19-related symptoms in the 4 weeks preceding the serological tests. However, only 9 (39.1%) of them had antibodies against SARS-CoV-2. In other words, 6 out every 10 infected otolaryngologists were asymptomatic. Bampoe et al. conducted a cross-sectional study at two maternity units in London and assessed 200 health-care workers, including anesthesiologists, obstetricians, and midwives in May and June of 2020. They observed that 59% of those infected (n = 29) had been asymptomatic or had very mild symptoms that did not meet the criteria for isolation. In the same study, anosmia was the only symptom predictive of seroconversion.^18^

Reports of headaches, fatigue, myalgia, and anosmia/ageusia differed significantly between seronegative and IgM and/or IgG positive participants (Table 2). Early on in the pandemic, changes to sense of smell and taste had already been robustly associated with COVID-19.^19^ Lechien et al. conducted a multicenter European study of 417 mild and moderate cases of COVID-19 and also reported a significant association with changes to sense of smell and taste.^20^

Approximately 95% of the otolaryngologists who participated in the present study work in clinics and/or hospitals, and all of them reported some degree of reduction in the number of patient appointments and surgical procedures performed during the study period. Nevertheless, 157 of the 358 participants were still seeing more than 40 patients per week during the study period. There were no statistically significant differences between the seropositive and seronegative groups in terms of number of patients appointments, proportion of patients with respiratory complaints, or type of hospital setting (whether taking COVID-19 referrals or not).

All participants reported having access to PPE. There was no statistically significant difference between the seropositive and seronegative groups in terms of access to the more complete standard (comprising N95/FFP2 mask + apron + gloves + face shield) or the more basic standard (N95/FFP2 mask + gloves). Many publications have addressed the use of PPE utilized by health-care workers. At the start of the pandemic, the WHO published instructions on the rational use of PPE^21^ and recommended wearing of surgical masks, gloves, eye protection, and aprons in cases that did not involve suspected or diagnosed COVID-19 and N95/FFP2 masks, gloves, aprons, head covering, and face shield for aerosol-generating procedures in confirmed cases of COVID-19. In May, the American Academy of Otolaryngology Head and Neck Surgery also published its recommendations, ranging from surgical masks, gloves, and eye protection in the care for patients with unknown or negative status in procedures with no aerosol generation, to N95 masks or powered air purifying respirators, eye protection, gloves, and aprons in cases involving mucosal manipulation. The same publication also highlighted that, at that point in time, there was not yet definitive evidence of transmission during otolaryngological procedures; only the anecdotal reports described above, which were later refuted.^22^

With regard to the risk factors associated with SARS-CoV-2 infection, the population-based study conducted by the state government/UFPel demonstrated that 33% of the family members of seropositive individuals were also positive for COVID-19,^11^, ^12^ similar to our prevalence of 34.8% of participants with infected household contacts, which was a statistically significant finding. Considering this in conjunction with the fact that no statistically significant association was found between the level of occupational exposure to infected patients and seropositivity, it can be speculated that most seropositive otolaryngologists were possibly infected outside the workplace. Although the timing of infections in the participants and their relatives is not known, this hypothesis is compatible with the findings of a cross-sectional study of IgG antibody detection in physicians, nurses, and other health-care workers at a Belgian hospital. The prevalence of positive cases was 6.4%, and neither clinical practice nor contact with patients with COVID-19 increased the risk of infection. However, presence of a household contact with suspected or confirmed SARS-CoV-2 infection increased by 3.15 times the risk of seropositivity.^23^ The authors suggested that the high availability of PPE and rigid protocols for infection prevention may have contributed to the low prevalence of SARS-CoV-2 infection. In another cross-sectional study, Sikkema et al. used data from whole-genome sequencing of SARS-CoV-2 from patients and health-care workers in 3 Dutch hospitals. The authors concluded that, although direct transmission in hospitals cannot be ruled out, their data do not support widespread nosocomial transmission as the source of infection in patients or health-care workers.^24^ Quoting recent preliminary data from a prospective study published by the United States’ Center for Disease Control and Prevention, Grijalva et al. highlighted the elevated rate of SARS-CoV-2 transmission between members of the same household, both adults and children.^25^

Regarding the diagnostic performance of the antibody test used in our study, we found high sensitivity and negative predictive value (100% for both) that exceed the rates declared by the manufacturer (Table 3). There was a statistically significant association between positive self-reported results of RT-PCR and IgG testing (with a trend towards significance for IgM), with a positive result in tests administered as part of the present study. However, this interpretation is subject to the limitation that the gold standard was defined as a self-reported positive PCR. Quan-Xin Long et al.^26^ showed the validity of serology as a late-stage diagnostic method for patients with COVID-19 (100% seroconversion 19 days after onset of symptoms). The significant association observed here between absence of symptoms and a positive antibody test result is compatible with their conclusion that serological testing can be useful in the diagnosis of asymptomatic patients.

Our study has some limitations. Except for the rapid antibody test results, all other variables were self-reported by the participants. The diagnostic power of the rapid antibody test may be susceptible to recall bias. Another potential limitation is inherent in the cross-sectional design of the study, which does not allow us to establish a cause-and-effect relationship, as in the case of the presence of infected household contacts and SARS-CoV-2 transmission.

## Conclusions

In a representative sample of 80.1% of all otorhinolaryngologists in the state of Rio Grande do Sul, southern Brazil, from August 1 to September 15, 2020, the SARS-CoV-2 infection rate was 6.4%. Compared with official data from the State Government/UFPel, seroprevalence was higher in otolaryngologists than in the general population in the same period. The rate of COVID-19 in all health-care workers in the state was 7.69% at the end of the same period. Using personal protective equipment, the level of occupational exposure did not result in higher rates of infection than other health professionals, but the presence of infected household contacts was associated with higher rates of seroconversion.

## Conflicts of interest

The authors declare no conflicts of interest.
